# Barriers and Facilitators to Workforce Changes in Integrated Care

**DOI:** 10.5334/ijic.3587

**Published:** 2018-06-01

**Authors:** Loraine Busetto, Katrien Luijkx, Stefano Calciolari, Laura Guadalupe González Ortiz, Hubertus Johannes Maria Vrijhoef

**Affiliations:** 1 Department of Neurology, Heidelberg University Hospital, Heidelberg, DE; 2 Tranzo Scientific Center for Care and Welfare, Tilburg University, Tilburg, NL; 3 Institute of Economics (IdEP), Università della Svizzera Italiana, CH; 4 Department of Patient and Care, Maastricht University Medical Center, Maastricht, NL; 5 Department of Family Medicine and Chronic Care, Vrije Universiteit Brussel, Brussels, BE; 6 Panaxea BV, Amsterdam, NL

**Keywords:** integrated care, chronic care, health workforce, quadruple aim, multimethod research, qualitative research

## Abstract

**Introduction:**

The aim of the study is to investigate the barriers and facilitators to the implementation of workforce changes implemented as part of integrated chronic care interventions.

**Methods:**

We used a qualitative multimethod design that combined expert questionnaires, a systematic literature review, and secondary analysis of two case reports. Twenty-five experts, twenty-one studies and two case reports were included in the study.

**Results:**

Most barriers related to problematic delivery structures, health professionals’ skills and enthusiasm, IT, funding, culture and cooperation and communication. Most facilitators related to health professionals’ motivation and enthusiasm, good delivery structures, communication and cooperation, IT, patients, leadership and senior management. Overall, similar categories of barriers and facilitators were found.

**Discussion:**

We recommend that future research focusses on more complex designs including multiple data sources, as these are better able to capture the complexity of interventions such as integrated care. We recommend that health managers and policy-makers should invest in delivery structures and skills and motivation of health professionals to improve the implementation of workforce changes in integrated chronic care interventions.

**Conclusion:**

The added value of the present study lies in its provision of information on which factors might mitigate the success of an intervention, which helps to prevent premature conclusions of ineffectiveness for complex interventions.

## Introduction

Health systems are faced with an ageing population and an increase in chronic conditions. These challenges require more appropriate approaches than the current largely single-disease and acute-care focussed health care systems. Integrated care is seen as one of the most promising of these approaches by targeting the health system, patient-provider relationships, care process design, communication infrastructures, community resources, and how care is delivered by health professionals [1,2,3,4]. In doing so, integrated care is expected to improve population health, patient experiences and cost-efficiency [5,6,7,8], a trio of goals commonly referred to as the Triple Aim [9].

Given health professionals’ involvement in all aspects of integrated care delivery, changes to the health workforce affect the implementation of integrated care profoundly. It has therefore been argued that the health workforce should be included as a fourth aspect in the Tripe Aim, thereby extending it to a Quadruple Aim of healthcare improvement [10]. By health workforce, we mean the clinical and non-clinical staff responsible for public and individual health intervention [11]. Workforce changes are those changes experienced by the health workforce, including for example nurse involvement, multidisciplinary staff, the introduction of multidisciplinary protocols, provider training, case managers, team meetings and the creation of new positions [12].

In a previous study on the outcomes of integrated care interventions including workforce changes, we found improvements in quality of care (including clinical patient outcomes and process measures), patient satisfaction and staff satisfaction [13]. However, improvements were not always found and it was not clear when and why successful outcomes could be achieved. We therefore called for more attention to be paid to the setting in which integrated care interventions are implemented. This call is in line with a growing body of work in implementation research that investigates the characteristics of implementers (i.e. those involved in and/or responsible for the implementation of an intervention in practice) that support or prohibit successful implementation processes [14]. For example, Greenhalgh et al. have called for increased focus on the processes by which particular health interventions are implemented and sustained in particular settings [15]. This type of research requires the use of complex research designs that focus on the barriers and facilitators to the implementation of an intervention, instead of simply focusing on observations before and after the introduction of the integrated care intervention and stripping away those factors expected to have a confounding effect on the causal relationship between intervention and outcome [16,17,18]. Additionally, complex designs should incorporate multiple data sources in order to include different perspectives of stakeholders to a specific intervention and triangulate results in explicit ways, which contributes to the methodological quality of the research [19,20,21].

The aim of this study was therefore to investigate the barriers and facilitators to the implementation of workforce changes implemented as part of integrated care interventions for people with chronic conditions. To this purpose, we used a qualitative interactive design with multiphase combination timing including a qualitative expert questionnaire, systematic literature review, and secondary analysis of two case reports. This study has an international scope and was conducted as part of Project INTEGRATE on “Benchmarking Integrated Care in Chronic and Age-related Conditions in Europe”, financed by the European Commission. Project INTEGRATE aimed to investigate the leadership, management and delivery of integrated care to help European care systems responding to the challenges of an ageing population and the increasing number of people living with chronic conditions.

## Methods

We used an interactive multimethod design with multiphase combination timing, which is characterised by direct interaction between different data strands and a combination of concurrent and sequential timing [22]. Concurrent timing refers to the collection of data strands during a single phase of the study, whereas sequential timing refers to data collection in two distinct phases, where one phase is carried out after the other. Specifically, data collection took place concurrently, data analysis sequentially, and the results of the analysis of one data source was used as the basis for the analysis of the next data source. Ethical approval was not required under Dutch law. A detailed overview of the workforce changes for whose implementation the barriers and facilitators are described in this manuscript is provided elsewhere [12].

### Definitions

In line with previous research, interventions were considered integrated care when targeting more than one Chronic Care Model (CCM) component (i.e. health system, self-management support, delivery system design, decision support, clinical information system and community) [2,24,25,26]. By barriers and facilitators we mean those factors that either hinder or foster the implementation or execution of integrated care interventions in practice. It should be noted that these factors can act as both barriers and facilitators [27].

### Data collection and analysis

When implemented as part of integrated care interventions, workforce changes are not an independent intervention, but one aspect of a complex intervention that also includes other aspects. This means that barriers and facilitators can be reported specifically for the *workforce changes* included in the integrated care intervention, or for the *overall integrated care intervention* that includes workforce changes. The distinction is that the former approach focusses specifically on one aspect of a complex intervention, namely workforce changes, whereas the latter focusses on the complex intervention as a whole, namely integrated care. We combined both of these approaches in this study: via expert questionnaires, we measured the factors affecting the workforce changes, via a literature review the factors affecting the overall intervention, and via case reports the factors affecting the overall intervention but with specific focus on workforce-related aspects of the integrated care intervention. The latter includes those aspects that affect clinical and non-clinical staff involved in the intervention and are, for example, related to the changes experienced by the health workforce. The research design is reported in detail elsewhere [12,23]. A description of the main elements of the data collection and analysis is provided in the following.

#### Expert questionnaires

Between January and April 2015, we administered a qualitative exploratory questionnaire to experts in the fields of integrated care, chronic care, and health human resource management. We included experts with academic or policy backgrounds as well as ‘field experts’ (i.e. health professionals or managers of organisations involved in the provision of integrated care). Given the study’s international scope, the questionnaire was translated from English to Dutch, Italian, and Spanish, based on a feasible adaption of recommendations provided in the relevant scientific literature [28,29,30,31], including a check by an English native speaker, forward translations by native speakers of the target language, back translations to English by a researcher proficient in English, and a discussion of the English versions. Experts were recruited using the snowball method. In the questionnaire, experts were asked to describe an integrated chronic care intervention, the workforce changes implemented as part of this intervention as well as the barriers and facilitators encountered in the implementation of these workforce changes. These were open questions to which free-text answers were expected. Due to the intended international scope and exploratory nature of the research, we opted for a questionnaire in order to increase our reach, instead of, for example, interviews or focus groups that would allow for gathering more in depth data, but would limit the range of experts that could potentially be included in the study. Furthermore, we only provided a written version of the questionnaire, as we did not expect that conducting the questionnaire by mail or telephone would contribute to increasing the response rate as respondents may have been unwilling or unable to pay the postage or speak a non-native language over the phone [32].

During an open coding phase, two researchers (LB, KL) independently created coding lists. During the subsequent axial and selective coding phases, the coding lists were compared and consolidated after discussion among the researchers (LB, KL). Examples based on the data were added for each code.

#### Literature Review

**Figure 1:**
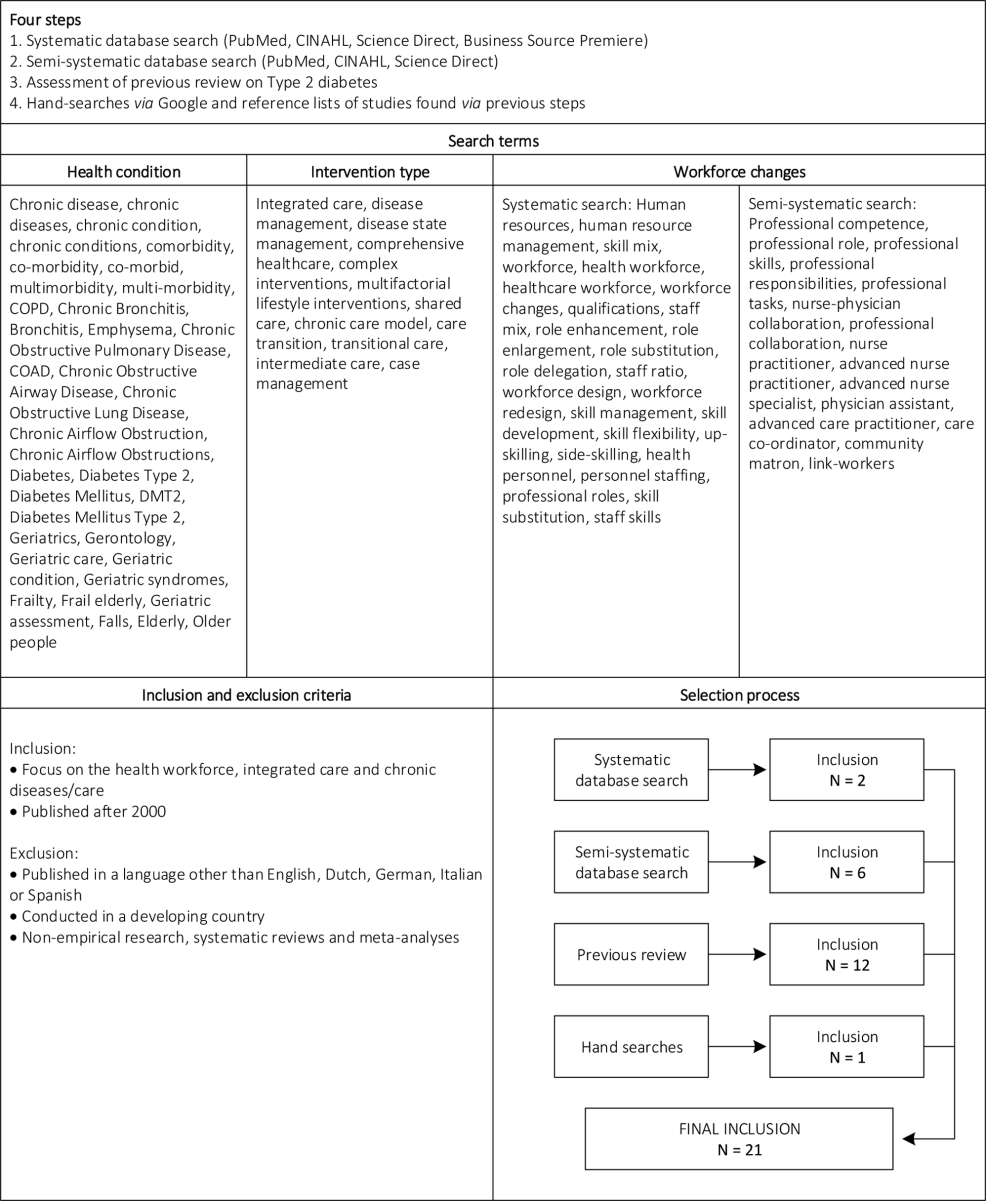
Summary of the literature review. *Notes:* Developing countries were defined following the World Economic Situation and Prospects (WESP) classification used by the United Nations.

Between July and October 2014, a literature search was conducted, including a systematic database search, a semi-systematic database search, unsystematic hand searches and secondary analysis of a previous literature review [1]. Figure 1 shows a summary of the literature review, including databases, search terms, inclusion and exclusion criteria, and the selection flowchart. In the systematic database search, articles were assessed individually by three researchers (LB, SC, LG) and then discussed together until consensus was reached. After the systematic database search yielded only a limited number of studies (N = 2), these preliminary results were discussed at a Scientific Committee Meeting of Project INTEGRATE. After consultation with the committee members, another set of health workforce related search terms was compiled and added to the search (see Figure 1). The new results were assessed in a semi-systematic way, meaning that one researcher performed the title and abstract selection, but full-text versions suggested for inclusion were discussed by three researchers (LB, SC, LG). As a third step, studies from a previous review that applied the same criteria regarding integrated care and chronic conditions but had a focus on one specific chronic condition (namely type 2 diabetes) were checked for a focus on health workforce changes by one researcher (LB) and included if applicable. Fourth, hand-searches were conducted on reference lists and via Google searches.

The data extraction was performed independently by three researchers (LB, SC, LG) and compared in pairs of two until consensus was reached (LB and SC, LB and LG, SC and LG). While in the expert questionnaires, experts were asked specifically about the workforce changes included in the integrated care interventions, for the studies data was extracted on the barriers and facilitators to the overall intervention because the studies reported integrated care interventions without specific focus on the workforce changes. The data were coded based on the coding lists resulting from the analysis of the expert questionnaires. Two researchers (KL, LB) performed the coding independently and discussed the results together until consensus was reached. When the data did not fit the categories of the coding lists, the lists could be adapted accordingly.

#### Case reports

Two detailed case reports of integrated care interventions implemented in the Netherlands and Germany were available for secondary analysis [33,34]. The Dutch case study concerned integrated care for people with type 2 diabetes in the primary care setting, including care groups, bundled payments, patient involvement, health professional cooperation, task substitution, evidence-based care protocols and a shared clinical information system [12,35,36]. The German case study concerned integrated care for geriatric conditions in a secondary care setting. Here, integrated care included a specific reimbursement system called “early complex geriatric rehabilitation”, multidisciplinary cooperation and comprehensive geriatric assessments [12,35,37]. Both case reports provided data on barriers and facilitators, but these were not specifically linked to the workforce changes of the integrated care intervention. This additional step was performed by one researcher (LB) during the secondary analysis by assessing whether the reported barriers and facilitators themselves involved workforce aspects or whether they were related to or impacted on workforce aspects of the respective integrated care intervention. The barriers and facilitators described in the case reports were coded using the coding lists from the analysis of the expert questionnaires and literature review. The coding was performed independently by two researchers (LB, KL) and discussed in pairs until consensus was reached. When the data did not fit the categories of the coding lists, the lists could be adapted accordingly.

#### Synthesis

After analysing the barriers and facilitators per data source, we compared those barriers and facilitators that were among those mentioned by most experts, studies or cases. For the literature review and expert questionnaires, we included barriers and facilitators that belonged to the three highest percentages per data source. Given the low number of case reports, we only included those barriers and facilitators that were present in both case reports.

## Results

We first present general information for each data source, before presenting the results on the barriers and facilitators.

### General information

#### Expert questionnaires

The questionnaires were sent to 91 experts and returned by 25, resulting in a response rate of 28%. Examples of integrated care interventions included the integration of primary, community and social care services, multidisciplinary teams, comprehensive geriatric assessments, provider education in self-management support, and comprehensive care trajectories. Interventions were implemented in 12 different countries, including Belgium (N = 8), Spain (N = 5), Estonia (N = 2), Italy (N = 2), the Netherlands (N = 2), the United Kingdom (N = 2), Australia (N = 1), Czech Republic (N = 1), Germany (N = 1), Greece (N = 1), Norway (N = 1), and Switzerland (N = 1).

#### Literature review

The final selection consisted of 21 studies [38,39,40,41,42,43,44,45,46,47,48,49,50,51,52,53,54,55,56,57,58]. Examples of integrated care interventions included patient education by specialist nurses, structured patient-oriented care coordination, the use of a patient registry to support multidisciplinary team work and practice nurse involvement in shared medical appointments. The studies described interventions implemented in seven countries, including the United States (N = 10), the Netherlands (N = 4), the United Kingdom (N = 2), Canada (N = 2), Belgium (N = 1), Austria (N = 1), and Germany (N = 1).

#### Case reports

The German case study was conducted at a geriatric hospital where each ward is organised in independent interprofessional teams consisting of doctors, physiotherapists, occupational therapists, nurses and neuropsychologists. A scientific paper based on this report is publised elsewhere [37]. The Dutch case study was conducted among two care groups implementing integrated care for people with type 2 diabetes. Care groups are legal entities that establish contracts with health insurers and health professionals to coordinate the so-called ‘care chain’ of chronic care from diagnosis to after care [59]. A scientific paper based on the detailed report is published elsewhere [36].

### Barriers & Facilitators

#### Expert questionnaire

Barriers and facilitators to the implementation of the workforce changes were found for the following 17 categories (in alphabetical order):

- Communication and cooperation, i.e. how health professionals communicate and cooperate with each other and with their patients;
- Competition, priority, pressure, i.e. the competition, (competing) priorities and external pressures experienced by health professionals;
- Complexity, i.e. the complexity of the intervention and/or daily practice experienced by health professionals;
- Culture, i.e. similarities or differences in the cultures in which the health professionals act and implement the integrated care interventions;
- Delivery structures, i.e. the structures of the delivery system in which the integrated care intervention is being implemented;
- Funding, i.e. the funding provisions for the implementation of the integrated care intervention;
- Health and social care, i.e. factors related to the health and social care sectors that affect the implementation of the integrated care intervention;
- Health professionals, i.e. intrinsic factors related to health professionals’ motivation and enthusiasm;
- Implementation support, i.e. factors related to how the implementation of the integrated care intervention is internally and/or externally supported;
- Incentives, i.e. incentives or disincentives targeted at and/or actually affecting the implementation of an integrated care intervention;
- Information technology (IT), i.e. the information technology infrastructure and devices related to the implementation of the integrated care intervention;
- Leadership and senior management, i.e. the leadership and senior management involved in and affecting the implementation of the integrated care intervention;
- Patients;
- Political and health system; i.e. factors related to the macro-level political and health system that affect the implementation of the integrated care intervention;
- Primary and secondary care; i.e. factors related to the primary and secondary care sectors that affect the implementation of the integrated care intervention;
- Time; i.e. factors related to time (constraints) affecting the implementation of the integrated care intervention;
- Other.

The barriers mentioned by most respondents related to delivery structures and health professionals (N = 11; 44%), followed by culture (N = 10; 40%), and funding (N = 8; 32%). Delivery structure barriers related to nurse-led care, guidelines and checklists, staffing and delineation of responsibilities. As regards the delineation of responsibilities, examples of barriers included arguments about the responsibilities of primary vs. hospital care and the lack of clarity of who should execute the intervention (general practitioner (GP), nurse, or someone else). With regard to staffing, barriers related to the number of specialised geriatric doctors or nurses, the difficulty of attaining and maintaining a sufficient number of staff and a high staff turnover. Health professional barriers related to their lack of knowledge, skills and expertise, education, enthusiasm and support, and other factors. Examples included the lack of knowledge on how to execute the intervention, worries around competencies and abilities, lack of engagement, worries about an additional (administrative) burden, the fear of change, and the fear of making mistakes or having them made public.

The facilitators mentioned by most respondents related to health professionals (N = 13; 52%), followed by leadership and senior management (N = 10; 40%), and delivery structures, patients, and communication and cooperation (N = 7; 28%). With regard to health professional facilitators, most respondents mentioned facilitators related to their enthusiasm and support, including examples such as enthusiastic professionals and volunteers, existence of a health professional championship to raise confidence for the program, and the fact that staff involvement led to enthusiasm which led to more cooperation and better implementation. Provider education facilitators included examples such as management involvement in the process to send staff to courses, education of interprofessional teams, and the existence of two regional laws that promote education activities. Facilitators related to leadership and senior management included strong and committed leadership, support from senior management, and good communication with hospital management.

#### Literature Review

Barriers and facilitators to the implementation of integrated care interventions including workforce changes were found for 14 of the 17 categories from the expert questionnaire. The three categories for which the studies did not report barriers and facilitators were complexity of the intervention, primary and secondary care, and health and social care. It was not necessary to adapt the coding lists.

The barriers described in most studies related to delivery structures (N = 7; 34%), health professionals (N = 6; 29%), IT (N = 5; 24%) and communication and cooperation (N = 5; 24%). Most delivery structure barriers related to staffing and included examples such as insufficient staff capacity, high staff turnover, cost of hiring sufficient staff, and an appropriate salary. Most barriers within the category health professionals related to health professionals’ knowledge, skills and expertise and included perceived lack of expertise, problematic understanding and implementation of diabetes education, and the fact that doctors were uncomfortable with computers and e-communication. Communication and cooperation barriers included drawbacks to standardised communication (e.g. the risk that the context of the data collection is lost from view and that the tone of the communication is misconstrued), perceived unwillingness to share care, and barriers to building a registry (e.g. multiple data sources, inconsistent formatting, unwillingness to share data).

The facilitators described in most studies related to delivery structures (N = 6; 29%), health professionals (N = 6; 29%), IT (N = 5; 24%), and patients (N = 4; 19%). Examples of facilitators relating to delivery structures included the use of a multidisciplinary team, systematic identification and assignment of patients and weekly case conferences. Within the category health professionals, most facilitators related to provider education and included continuous education efforts, extended training periods and participatory methods, and offering staff continuous education credit. Examples of IT facilitators included a registry, a social networking module that allowed participants to connect with each other, and mandatory, pre-structured patient files on the internet. Patient facilitators related to patient centeredness and involvement and support, awareness and motivation. Examples included the collaborative development of self-care plans reflecting both treatment indications and patient preference, a home visit tutorial conducted by the project staff, less formal diabetes education, and behavioural goal setting.

#### Case reports

Barriers and facilitators were found for 13 categories. The coding list did not need to be adapted. Both case reports reported barriers for the categories delivery structures, health professionals and IT (N = 2; 100%). Delivery structure barriers included staff shortages, strict guidelines that gave health professionals very little room to adapt the requirements to the specific needs of the patients, and the fear that tasks would be taken away when new professional roles were introduced. Most health professional barriers related to their lack of enthusiasm and support and included examples such as resistance to the introduction to integrated care by GPs and frustration caused by too many innovations within a short time frame. IT barriers included strict data protection frameworks and the separation of data systems between different health sectors or primary and secondary care.

Facilitators were reported in both case reports for the categories delivery structures, health professionals, IT and communication and cooperation (N = 2; 100%). Examples of delivery structure facilitators included the facilitating role of a practice nurse in the cooperation between the GP and other health professionals, and organisational workarounds to compensate for the lack of official structures and tools to support multidisciplinary care delivery. Health professional facilitators included the personal experience and competences of the clinical leader and founder of a geriatric hospital, the existence of a group of innovators to drive innovations, and the increased conviction by health professionals that integrated care helps to improve the quality of care.

#### Synthesis

Table 1 shows the barriers that were mentioned most often. Delivery structure and health professional barriers were among the three highest percentages in the expert questionnaire and literature review, and mentioned in both case reports. IT barriers were among the three highest percentages in the literature review and mentioned in both case reports. Funding and culture barriers were among the three highest percentages in the expert questionnaires and communication and cooperation barriers among the three highest percentages in the literature review.

**Table 1:** Overview of the barriers mentioned by most experts, studies or cases.

Barriers	Expert Questionnaire (N = 25)	Literature Review (N = 21)	Case Reports (N = 2)

Delivery structures	**44%**	**34%**	**100%**
Health professionals	**44%**	**29%**	**100%**
IT	16%	**24%**	**100%**
Funding	**32%**	14%	50%
Culture	**40%**	10%	0%
Communication and cooperation	4%	**24%**	0%

*Note:* Percentages in bold print indicate that the respective barrier was among the three highest percentages in the expert questionnaire or literature review, or mentioned in both case reports. Percentages in normal print indicate that the respective barrier was not among the three highest percentages in the expert questionnaire or literature review, or mentioned in both case reports.

Table 2 shows the facilitators that were mentioned most often. Facilitators related to health professionals and delivery structures were among the three highest percentages in the expert questionnaires and literature review, and reported in both case reports. Facilitators relating to communication and cooperation, IT and patients were mentioned most often in two of the three data sources. Leadership and senior management facilitators were among the three highest percentages in the expert questionnaires.

**Table 2:** Overview of the facilitators mentioned by most experts, studies or cases.

Facilitators	Expert Questionnaire (N = 25)	Literature Review (N = 21)	Case Reports (N = 2)

Health professionals	**52%**	**29%**	**100%**
Delivery structures	**28%**	**29%**	**100%**
Communication and cooperation	**28%**	14%	**100%**
IT	8%	**24%**	**100%**
Patients	**28%**	**19%**	0%
Leadership and senior management	**40%**	5%	50%

*Note:* Percentages in bold print indicate that the respective facilitator was among the three highest percentages in the expert questionnaire or literature review, or mentioned in both case reports. Percentages in normal print indicate that the respective facilitator was not among the three highest percentages in the expert questionnaire or literature review, or mentioned in both case reports.

## Discussion

This study has described the barriers and facilitators to the implementation of workforce changes in integrated chronic care interventions. To this purpose, three methods of data collection were combined, namely expert questionnaires, a literature review and case reports.

Our research adds to a considerable body of existing research about implementing health care interventions. For example, in their study on how to foster implementation of health services research findings into practice, Damschroder and colleagues have developed and described a Consolidated Framework for Implementation Research [60]. This framework includes five major domains that include intervention characteristics, outer setting, inner setting, characteristics of the individuals involved, and the process of implementation. Our findings are reflected in all of these domains (e.g. complexity and cost in intervention characteristics; external pressures and political and health system in outer setting; delivery structures, communication, cooperation, culture, competition, priority and leadership in inner setting; patient in characteristics of the individuals involved, and health professional championship in the process of implementation). We did not see our IT category reflected in the Damschroder framework. Additionally, Greenhalgh et al. report a meta-narrative literature review conducted to answer the question of how innovations can be spread and sustained in health service delivery and organisation [15]. In doing so, they developed a unifying conceptual Model of Diffusion in Service Organisations whose main aim is to serve as a memory aide for considering the different aspects and interactions in a given complex situation. Again, our findings are reflected in this model, too (not the least because the Model of Diffusion in Service Organisations was one of the models on which the Consolidated Framework for Implementation Research was based). What is common to these approaches is the assumption that it is not enough to only focus on the outcomes of an intervention, but that, instead, the implementation process and how this influences the intervention and its outcomes, is at least equally important. Or as Durlak and DuPre phrase it in their review of research on the influence of implementation on program outcomes and the factors affecting implementation: “implementation matters” [14]. This is also reflected in the WHO Europe’s report on strengthening a competent health workforce for the provision of coordinated/integrated health services [61]. In addition to outlining the competencies that are essential for a successful health workforce, the report stresses the importance of environments and actions that facilitate the execution of existing competencies as well as the acquisition of new competencies throughout the so-called competency consolidation cycle. This is reflective of the assumption of this paper that initial acquisition of competencies or initial implementation of workforce interventions is only the beginning of a long-term, iterative process, often in the form of cycles that may backtrack to the start. We have added to this body of knowledge by providing insights into the barriers and facilitators to workforce changes in integrated care specifically.

We found most barriers to the implementation process to be related to delivery structures, including problematic staffing and delineation of responsibilities. Health professionals’ lack of skills and enthusiasm were also perceived as problematic. Barriers related to IT, funding, culture and communication and cooperation were also reported. Barriers such as high costs, problematic funding, lack of health professionals’ motivation and technical barriers were also reported in a recent European study on the continuous professional development of different health professions [62]. Furthermore, a study on team training and organisational change in the Canadian context found barriers relating to the resistance to share care, high costs, and staffing problems [63]. A review exploring the characteristics of effective workforce practice in integrated health and social care services found difficulties in information sharing to be an important barrier [64]. Finally, a scoping study by the British National Health Service (NHS) also found gaps in the skills of staff who provide integrated care for older adults [65].

Most facilitators were related to health professionals’ motivation and enthusiasm. Good delivery structures including a sufficient number of staff and nurse-led care also helped the implementation process. Other facilitators concerned communication and cooperation, IT, patients and leadership and senior management. Health professionals’ motivation was also identified as a facilitator in a review on the changing skill-mix of the health workforce [66]. A study on multidisciplinary meetings in geriatric assessment units found nurse involvement to be a facilitator of multidisciplinary collaboration [67]. The study on team training and organisational change in the Canadian context referenced above found leadership support and health professionals’ willingness to change to be important facilitators for workforce change. Good leadership was also identified as an important facilitator by the review on characteristics of effective workforce practice in integrated health and social care services mentioned above, together with provider education, a clear understanding of different roles and responsibilities, and good communication between professionals [64]. A recent European study on new professional roles in health care found established health professionals’ willingness to change and new roles that emphasise patient-centred care to be important facilitators as well [68]. The importance of staff engagement in the implementation of workforce changes was also outlined in a recent study from England [69]. Additionally, patient engagement was identified by the NHS as a facilitator for responsive and adaptive integrated care services [65]. Finally, the use of IT systems was identified as an important facilitator for better health human resource utilisation and empowerment of elderly patients [70].

As mentioned above, when implemented as part of integrated care interventions, workforce changes are not an independent intervention, but only one aspect in a complex intervention. Because of these different emphases on one aspect of an intervention as opposed to the overall intervention, one would expect the literature review to reveal barriers and facilitators for the highest number of different categories, because of its broad focus on the overall intervention, as opposed to the expert questionnaire, which had a more narrow focus on the workforce changes within the overall intervention. However, as mentioned above, the experts reported barriers and facilitators for 17 categories, while the studies only reported 14 categories, leaving out the categories complexity of the intervention, primary and secondary care, and health and social care. This difference may be explained by the fact that especially the latter two categories can be more easily witnessed by an expert observer than systematically measured as would be appropriate for published academic literature. Another reason could be that these factors have received attention in academic publications only relatively recently and might therefore not come to the fore in systematic reviews of the literature. The case reports form an exception because they focussed on only one intervention each. Therefore, it is not surprising that the case reports only found barriers and facilitators for 13 categories, even though they had a relatively broad focus on the overall intervention with a focus on workforce-related issues. Despite these differences, the 17 categories found via the expert questionnaire did not need to be extended for the literature review and case reports. This shows that, overall, the three approaches yielded similar findings.

It is also noticeable that similar categories of barriers and facilitators were found. This points toward the fact that most factors can act as facilitators as well as barriers, depending on whether they are present or absent or how well they are implemented. For example, the lack of an IT system can be a huge barrier to efficient care delivery, but so can an IT system that creates more problems than it was intended to solve. Faced with such a problematic IT system, the lack or circumvention of an IT system would be a facilitator, but of course, so would be an IT system that works well. In this sense, barriers and facilitators are often two sides of the same coin. Additionally, barriers and facilitators are often causes and consequences of one another. To use the previous example, a health IT system might be implemented to facilitate the communication between health professionals working at different locations and to standardise information exchange. However, when replacing face-to-face or phone contact, digitalised and standardised communication can act as a barrier to good working relationships between the different health professionals. This might lead to the implementation of new solutions which, in turn, might cause new problems. This interrelatedness of barriers and facilitators reflects the complexity that is inherent to integrated care interventions and shows that the implementation of most complex interventions is never really complete. More attention should therefore be paid to the interplay between different factors as well as the levels at which this interplay occurs. A useful framework for these types of analyses is the PARIHS framework for promoting action on research implementation in health services [71,72]. The findings of this study represent the first steps in the identification of complex relationships and thereby pave the way for further reflection and possible disentanglement by stakeholders such as academic experts and health and social care professionals who should be involved in future research on this topic.

There are methodological limitations to this study which should be taken into consideration. First, the interpretation of answers to the expert questionnaire was sometimes ambiguous. While some answers were very detailed and informative, others were too short to make sense of or were so ambiguous that they generated more questions than they answered. When one of those answers (e.g. “IT” or “no money”) matched a category rather clearly (e.g. the categories “IT” or “Funding”), we mapped the answer to the respective category. However, when there was no clear link between a short or ambiguous answer and an already existing category, the answer was excluded from the analysis. Future research should make use of data collection methods that allow for the collection of more in depth data such as interviews and focus groups. Second, we summarised the findings from the different sources by reporting whether the respective barriers or facilitators were among the three highest percentages in the expert questionnaire or literature review, or mentioned in both case reports. Of course, frequent reporting of barriers and facilitators does not necessarily mean that they are the key factors affecting the implementation process. Although the frequency might suggest managers to consider such factors before the implementation process, further research is needed to understand and quantify the effect of barriers and facilitators on outcomes. A better understanding of the influence of barriers and facilitators would especially be supported by the inclusion of qualitative methods such as interviews and focus groups in future research, as suggested above. Third, the systematic part of our literature review identified only a very small evidence base on workforce changes. This is in line with previous studies on health workforce changes [64,65,66]. We tried to alleviate this shortcoming by adding semi- and unsystematic searches as well as consulting a previous literature review on diabetes. The latter revealed a rich evidence base on workforce changes in integrated care interventions even though its search strategy was not primarily concerned with workforce related search terms. This shows that not being able to find a large evidence base does not necessarily mean that it does not exist, but that it is not easily accessible. This could be the case because those who contribute to creating the scientific evidence on the topic might not realise that they are doing so because they are only focusing on a specific professional field or disease. The field would benefit from a common and clearly described terminology for workforce changes, but also from increased attention to a cross-cutting issue such as workforce changes that transcends those diverse and much specialised fields of interest.

One of the main strengths of this study lies in its international scope by including data from 15 different countries. This is especially relevant because there is no consensus definition of integrated care and the concept often has different meanings in different countries or health systems [73,74]. Moreover, the research plan as well as preliminary findings were regularly fed back to the scientific committee of Project Integrate, which enhanced the methodological quality of the study and ensured its relevance for the international academic and practice community [75]. Finally, the study combined three different methods of data collection in an interactive design with multiphase combination timing, which enabled the triangulation of findings. The added value of the present study lies in its provision of information on which factors might mitigate the success of an intervention, which helps to prevent premature conclusions of ineffectiveness due to inappropriate research designs for complex interventions [76,77]. Moreover, knowing which factors are conducive to successful implementation makes it possible to adequately inform policy-makers and practitioners regarding their choices for efficient allocation of scarce health resources. In the long run, this is expected to benefit the population with or at risk of chronic conditions as well as the health workforce caring for them.

## Conclusion

We recommend that future research focusses on more complex designs including multiple data sources and that researchers and practitioners pay more attention to context factors in evaluations of complex interventions, rather than focussing on outcomes only [71,72]. This would yield valuable information on the strengths and weaknesses of the execution of an intervention in a specific real-world setting and thereby increase the likelihood of implementing interventions successfully. Finally, we recommend for health managers and policy-makers to allocate significantly more resources to improving delivery structure and work environments of health professionals, as most barriers as well as facilitators were related to the number of health professionals, their skills, enthusiasm and division of tasks. These long-term investments into the health workforce, rather than short-term cost-cutting measures at the expense of the health workforce, are expected to contribute to the health and experiences of the population.
